# Crucial Role of Rapgef2 and Rapgef6, a Family of Guanine Nucleotide Exchange Factors for Rap1 Small GTPase, in Formation of Apical Surface Adherens Junctions and Neural Progenitor Development in the Mouse Cerebral Cortex^1^,^2^,^3^

**DOI:** 10.1523/ENEURO.0142-16.2016

**Published:** 2016-06-23

**Authors:** Kazuhiro Maeta, Hironori Edamatsu, Kaori Nishihara, Junji Ikutomo, Shymaa E. Bilasy, Tohru Kataoka

**Affiliations:** Department of Biochemistry and Molecular and Biology, Division of Molecular Biology, Kobe University Graduate School of Medicine, Kobe 650-0017, Japan

**Keywords:** Rapgef2, Rapgef6, Rap1 small GTPase, neural progenitors, neocortex development, adherens junction

## Abstract

Cerebral neocortex development in mammals requires highly orchestrated events involving proliferation, differentiation, and migration of neural progenitors and neurons. Rapgef2 and Rapgef6 constitute a unique family of guanine nucleotide exchange factors for Rap1 small GTPase, which is known to play crucial roles in migration of postmitotic neurons. We previously reported that conditional knockout of *Rapgef2* in dorsal telencephalon (*Rapgef2*-cKO) resulted in the formation of an ectopic cortical mass (ECM) resembling that of subcortical band heterotopia. Here we show that double knockout of *Rapgef6* in *Rapgef2*-cKO mice (*Rapgef2/6*-dKO) results in marked enlargement of the ECM. While *Rapgef2*-cKO affects late-born neurons only, *Rapgef2/6*-dKO affects both early-born and late-born neurons. The *Rapgef2*-cKO cortex at embryonic day (E) 15.5, and the *Rapgef2/6*-dKO cortex at E13.5 and E15.5 show disruption of the adherens junctions (AJs) on the apical surface, detachment of radial glial cells (RGCs) from the apical surface and disorganization of the radial glial fiber system, which are accompanied by aberrant distribution of RGCs and intermediate progenitors, normally located in the ventricular zone and the subventricular zone, respectively, over the entire cerebral cortex. Moreover, intrauterine transduction of Cre recombinase into the *Rapgef2^flox/flox^* brains also results in the apical surface AJ disruption and the RGC detachment from the apical surface, both of which are effectively suppressed by cotransduction of the constitutively active Rap1 mutant Rap1^G12V^. These results demonstrate a cell-autonomous role of the Rapgef2/6-Rap1 pathway in maintaining the apical surface AJ structures, which is necessary for the proper development of neural progenitor cells.

## Significance Statement

Rapgef2 is known to play a critical role in multipolar–bipolar transition of postmitotic neurons from shRNA-mediated knock-down experiments. This function of Rapgef2 was presumed to account for the formation of an ectopic cortical mass observed in *Rapgef2* conditional knock-out (*Rapgef2*-cKO) mice. In this article, by using *Rapgef2*-cKO mice and the intrauterine electroporation method, we are able to demonstrate a novel role of the Rapgef2/Rap1 pathway in the proper development of neural progenitors, particularly RGCs, which is presumably accounted for by its cell-autonomous role in maintaining the apical surface adherens junction structures. Moreover, we show that Rapgef6, a close Rapgef2 homolog implicated in the etiology of schizophrenia, shares some of these functions with Rapgef2 from the examination of *Rapgef2/6* double-knockout mice.

## Introduction

One of the hallmarks of the mammalian cerebral neocortex is that neurons with distinct morphology and functions are organized into six horizontal layers (Defelipe, 2011). In mice, layer formation occurs between embryonic day 11 (E11) and E18 in an inside-out manner, where waves of postmitotic neurons migrate successively through the previously formed layers (Hoerder-Suabedissen and Molnár, 2015). During neurogenesis, radial glial cells (RGCs), located in the ventricular zone (VZ), function as neural progenitors and generate self-renewing RGCs and neurons as well as committed intermediate progenitor cells (IPCs). IPCs move to the subventricular zone (SVZ), divide and differentiate into neurons. Newly born neurons adopt multipolar morphology and migrate to the intermediate zone (IZ), where they undergo multipolar–bipolar transition to assume bipolar morphology and migrate along the radial glial (RG) fibers to the cortical plate (CP), a process called RG fiber-guided locomotion. Finally, they undergo RG fiber-independent somal translocation to reach their final destinations, a process called terminal translocation. Concurrently, the somata of RGCs undergo radial translocation throughout the width of the VZ, depending on the phases of the cell cycle; the somata of S-phase and M-phase cells are located at the basal and apical sides, respectively. This confers to the VZ a false stratified appearance. Each RGC possesses two RG fibers, a basal fiber, which reaches the pial surface and serves as a scaffold for neuronal migration; and an apical fiber, whose end foot establishes adherens junctions (AJs) with those of neighboring RGCs, and forms the apical surface of the telencephalon lining the ventricles.

Rap1, consisting of the two isoforms Rap1a and Rap1b, is a close homolog of Ras small GTPases, and plays pleiotropic functions controlling cell proliferation, adhesion, polarity, and endocytosis (Gloerich and Bos, 2011). In particular, it plays a pivotal role in regulation of the integrin-mediated and cadherin-mediated cell adhesion (Boettner and Van Aelst, 2009). Like other GTPases, Rap1 functions as a molecular switch by cycling between GTP-bound active and GDP-bound inactive forms. Their interconversion is reciprocally stimulated by guanine nucleotide exchange factors (GEFs) and GTPase-activating proteins (GAPs), where GEFs mediate Rap1 activation in response to various extracellular stimuli. There exist an array of GEFs for Rap1, including Rapgef1 to Rapgef 6, RasGEF1A to RasGEF1C, CalDAG-GEF1/3, and phospholipase Cε, which are regulated by distinct signaling mechanisms and are responsible for differential regulation of Rap1 activity in spatial, temporal, and cell type-specific manners (Gloerich and Bos, 2011). During the mouse cerebral neocortex development, Rap1 was shown to play crucial roles in neuronal migration, particularly in the multipolar migration, the multipolar–bipolar transition, and the terminal translocation, but not the RG fiber-guided locomotion (Voss et al., 2008; Franco et al., 2011; Jossin and Cooper, 2011; Sekine et al., 2012). In these cases, Rapgef1, also called C3G, was shown to be involved in Rap1 activation downstream of the signaling of Reelin-Reelin receptors Dab1-Crk/CrkL.

Rapgef2, also called RA-GEF-1 and PDZ-GEF1 (de Rooij et al., 1999; Liao et al., 1999, 2001), and Rapgef6, also called RA-GEF-2 (Gao et al., 2001), constitute a unique Rap1-GEF family that is characterized by the possession of the PSD-95/DlgA/zona occludens-1 (ZO-1) and Ras/Rap-associating domains. Through association with the GTP-bound forms of Rap1 and M-Ras at the Ras/Rap-associating domains, they are recruited from the cytoplasm to the Golgi complex and the plasma membranes, respectively, and cause Rap1 activation by the action of their CDC25-homology domains. We showed that dorsal telencephalon-specific *Rapgef2* conditional knock-out (*Rapgef2*-cKO) mice develop an ectopic cortical mass (ECM) resembling that of subcortical band heterotopia, suggesting a role for Rapgef2 in neocortical development (Bilasy et al., 2009). Recently, Rapgef2 was reported to play a crucial role in the multipolar–bipolar transition of postmitotic neurons (Ye et al., 2014).

In this study, we observe severe defects in the development of RGCs and IPCs, and in the formation of the apical surface AJs in the developing cerebral cortices of *Rapgef2*-cKO and *Rapgef2/Rapgef6* double-knockout (*Rapgef2/6*-dKO) mice, revealing a novel role of the Rapgef2/6–Rap1 pathway in neural progenitor cells.

## Materials and Methods

### Animals

*Rapgef2^flox/flox^* (*Rapgef2*-f/f) and *Rapgef^−/−^* mice were generated as reported previously (Yoshikawa et al., 2007; Bilasy et al., 2009). *Rapgef2*-cKO mice were generated by mating *Rapgef2*-f/f mice with *Emx1^cre/+^* mice as described previously (Bilasy et al., 2009). The *Rapgef6*-KO mice used in this study were *Rapgef2^flox/flox^*;*Rapgef6^−/−^* mice. *Rapgef2/6*-dKO mice were generated by mating them with *Emx1^cre/+^* mice. Genotypes were determined by PCR as described before previously (Yoshikawa et al., 2007; Bilasy et al., 2009). All of the animals had been backcrossed to C57BL/6JJcl mice (http://jaxmice.jax.org/strain/013636.html) more than eight times and maintained on 12 h light/dark cycles with free access to food and water. To obtain embryos, female mice were placed together with a male mouse overnight, and the following morning (∼10:00 A.M.) was defined as E0.5. Operations were performed under general anesthesia by intraperitoneal administration of dexmedetomidine (0.3 mg/kg; Maruishi Pharmaceutical), midazolam (4 mg/kg; Astellas Pharma), and butorphanol tartrate (5 mg/kg; Meiji Seika Pharma) as described previously (Nagano et al., 2014). The use and care of the animals were reviewed and approved by the Institutional Animal Care and Use Committee of Kobe University.

### Antibodies

The following primary antibodies were used for indirect immunofluorescence staining: anti-Cux1 (sc-13024, Santa Cruz Biotechnology); anti-Ctip2 (ab18465, Abcam); anti- GFP (GFP-1010, Aves Labs); anti-Pax6 (PD022, MBL); anti-bromodeoxyuridine (BrdU; catalog #555627, BD Biosciences); anti-phospho-histone-H3 (Ser10; catalog #09-797, Millipore); anti-Rapgef2 (Wei et al., 2007); anti-Rapgef6 (Yoshikawa et al., 2007); anti-β-catenin (catalog #610153, BD Biosciences); anti-N-cadherin (catalog #610920, BD Biosciences); anti-E-cadherin (catalog #610181, BD Biosciences); anti-afadin (catalog #ab90809, Abcam); anti-ZO-1 (catalog #61-7300, Life Technologies); anti-nestin (catalog #556309, BD Biosciences); anti-Tbr1 (catalog #ab31940, Abcam); and anti-c-Myc (catalog #23941-54, Nacalai Tesque). To detect immunoreactive signals, the following secondary antibodies were used: Alexa Fluor 488-conjugated anti-chicken Ig Y (catalog #703-545-155, Jackson ImmunoResearch); Alexa Fluor 647-conjugated anti-rabbit IgG (catalog #A21244, Life Technologies); CF647-conjugated anti-mouse IgG (catalog #20281, Biotium); CF488A-conjugated anti-IgG (catalog #20302, #20015, #20027, Biotium); CF488A anti-mouse IgG (Biotium); and CF555-conjugated anti-IgG (catalog #20231, #20038, #20233, Biotium). An Alexa Fluor 647-conjugated anti-Tbr2 antibody (catalog #51-4875, eBioscience) was also used.

### Plasmids

A Cre recombinase expression vector, pCAG-NLS-Cre (Tsumura et al., 2006), was provided by Dr. N. Suzuki (Mie University, Tsu, Japan). pCAG=EGFP, an expression vector of enhanced GFP (EGFP; Nishimura et al., 2014), was a gift from Dr. K. Nagata (Aichi Human Service Center, Kasugai, Japan). pCAG-FloxP-EGFP-N1, which conditionally expresses EGFP in cells harboring Cre recombinase (Shitamukai et al., 2011), and pCAG-EGFP-F, which expresses EGFP attached with a C-terminal CAAX motif, the farnesylation signal, were provided by Dr. F. Matsuzaki (RIKEN Center for Developmental Biology, Kobe, Japan). pCAG-Myc-Rap1^WT^ and pCAG-Myc-Rap1^G12V^, which express wild-type Rap1 and Rap1^G12V^ attached with an N-terminal human c-Myc epitope tag, respectively, were generated by cloning the respective cDNAs (Liao et al., 1999) into a BamHI cleavage site of a derivative of pCAG=MCS2 (Kawauchi et al., 2005).

### Immunofluorescence

Preparation of histologic samples and immunostaining were performed as described previously (Bilasy et al., 2009). Serial sections (6 µm thick) of paraffin-embedded specimens and serial sections (20 or 50 µm thick) of frozen specimens were cut on a sliding microtome (SM2000R, Leica) and a cryostat (CM1850, Leica), respectively. Immunofluorescently stained sections were observed under a confocal laser-scanning microscope (LSM510 META, Carl Zeiss), and, if necessary, the focus stacking technique (also known as *z*-stacking) was used to digitally combine multiple images taken at different focus distances of each section. Hematoxylin and eosin (H&E)-stained sections were observed under an AX80 microscope with a DP70 camera (Olympus).

### Assessment of cell distribution

Radial distributions of neural progenitors and neurons were quantified essentially as described (Franco et al., 2011). Briefly, the neocortex and ECM were separately divided into 10 laminar bins with equal thickness and width, and the cells in each bin were counted. The percentage of the cells present in each bin was obtained as follows: 100 × the number of cells in each bin/the total number of cells in the 10 bins.

### BrdU labeling

BrdU was dissolved in sterile saline solution and intraperitoneally injected into pregnant females at a dose of 100 µg/g body weight. To detect S-phase cells, brain specimens of their embryos were harvested 0.5 h later, fixed in 4% paraformaldehyde, and subjected to immunohistological detection of BrdU. To trace the progenitor fate, embryonic brains were harvested at various time points and analyzed as described above.

### *In utero* electroporation

*In utero* electroporation (IUE) was performed at E14.5 using an electroporator (CUY21 EDIT II, BEX Co, Ltd.) along with a pair of forceps-type electrodes as described previously (Saito and Nakatsuji, 2001). The lateral ventricle of an embryo received 1 µl of sterilized water containing 0.01% FastGreen dye and the following plasmids: pCAG-EGFP-F (0.01 µg/µl) for labeling cells with farnesylated EGFP; pCAG-NLS-Cre (0.1 µg/µl) and pCAG-FloxP-EGFP-N1 (0.5 µg/µl) for deleting *Rapgef2* in *Rapgef2*-f/f embryos; pCAG=EGFP (0.5 µg/µl) for labeling cells without *Rapgef2* deletion in *Rapgef2*-f/f embryos; and pCAG-NLS-Cre (0.05 µg/µl), pCAG-FloxP-EGFP-N1 (0.25 µg/µl), and either pCAG-Myc-Rap1^WT^, pCAG-Myc-Rap1^G12V^, or pCAG-Myc (0.7 µg/µl each) for testing the compensation by Rap1. Subsequently, electroporation was performed by 450 ms pulses of 30 V separated by 980 ms.

### Reproducibility and statistical analysis

Images shown are representatives of ones obtained from at least three biological replicates. For graphs, data are expressed as the mean ± SD derived from biologically independent replicates whose numbers are indicated in their figure legends. If the *p* value obtained by the unpaired two-tailed Student’s *t* test was <0.05, the difference was considered statistically significant.

## Results

### Effects of *Rapgef*2-cKO and *Rapgef2/6*-dKO on the cortical architecture

*Rapgef2*-cKO mice had been shown to develop severe brain malformations characterized by the formation of a large ECM lacking the layer structure beneath a thin neocortex retaining the typical six-layered structure, the interruption of the pyramidal cells in the hippocampal CA1 region, the enlargement of the lateral ventricles, and the agenesis of interhemispheric connections (Bilasy et al., 2009, 2011). The ECM extended throughout the rostrocaudal axis of the cerebral hemisphere. On the other hand, the brains of *Rapgef6*-KO mice failed to show any morphological abnormalities (Yoshikawa et al., 2007; Levy et al., 2015).

**Figure 1.**
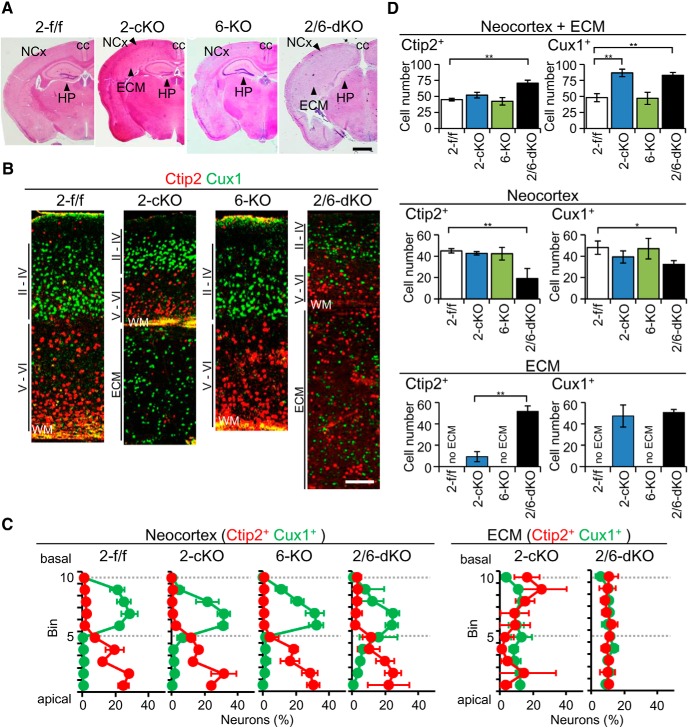
Effects of the genotypes of Rapgef2 and Rapgef6 on the cortical structure at P90. ***A***, H&E staining of the coronal sections of the brains of *Rapgef2*-f/f (*2-f/f*), *Rapgef2*-cKO (*2-cKO*), *Rapgef6*-KO (*6-KO*), and *Rapgef2/6*-dKO (*2/6-dKO*) mice. Representative specimens derived from six mice of each group are shown. The neocortex with the six-layered structure (NCx), hippocampus (Hp), corpus callosum (cc), and ECM are indicated. Scale bars, 2 mm. ***B***, Immunohistological staining of the neocortex and ECM. Sections prepared as in ***A*** were immunostained for Ctip2 (red) and Cux1 (green). Representative specimens derived from six mice of each group are shown. WM, White matter. Scale bars, 100 µm. ***C***, ***D***, Distributions of Ctip2^+^ and Cux1^+^ neurons in the neocortex and ECM. Sections stained as in ***B*** were used for the quantification of Ctip2^+^ neurons (red) and Cux1^+^ neurons (green), residing in each bin, as described in Materials and Methods (***C***), and the neurons contained in a total of 10 bins (100 μm in width) were counted as described in Materials and Methods (***D***). Data are expressed as the mean ± SD derived from six mice of each genotype. Student’s *t* test: *p < 0.05, **p < 0.01.

Considering the structural and functional similarity between Rapgef2 and Rapgef6, we first examined the effects of *Rapgef6* knockout on the brain malformations of *Rapgef2*-cKO mice by generating *Rapgef2/6*-dKO mice. At postnatal day 90 (P90), *Rapgef2/6*-dKO mice, compared with *Rapgef2*-cKO mice, showed more severe brain malformations, which are characterized by an enlarged ECM beneath a very thin neocortex and generalized atrophy of the hippocampus (Fig. 1*A*). Also, agenesis of the corpus callosum was evident. As reported before, the *Rapgef6*-KO brains did not show any morphological abnormalities compared with those of *Rapgef2*-f/f mice, which were used as a control throughout this study. Cortical layer structures were examined by immunostaining for Ctip2 and Cux1 to visualize early-born neurons in the layers V/VI, born between E12.5 and E13.5, and late-born neurons in the layers II/III and IV, born between E14.5 and E18.5, respectively (Fig. 1*B*,*C*; Greig et al., 2013). The thin neocortices of *Rapgef2*-cKO and *Rapgef2/6*-dKO mice kept an inside-out laminar organization as observed in the neocortices of *Rapgef2*-f/f and *Rapgef6*-KO mice. In contrast, their ECMs lost the laminar organization altogether. Neurons residing in the *Rapgef2*-cKO ECM were mostly composed of Cux1^+^ neurons, while those in the *Rapgef2/6*-dKO ECM were composed of a mixture of nearly equal numbers of Ctip2^+^ and Cux1^+^ neurons (Fig. 1*B*,*D*). Consistent with this, the *Rapgef2/6*-dKO neocortices showed a significant reduction in the numbers of both Ctip2^+^ and Cux1^+^ neurons, while the *Rapgef2*-cKO neocortices showed a reduction in the number of Cux1^+^ neurons only (Fig. 1*D*). These observations indicated that both early-born and late-born neurons were almost evenly affected by *Rapgef2/6*-dKO, whereas late-born neurons were predominantly affected by *Rapgef2*-cKO. The present results obtained with *Rapgef2*-cKO mice were indistinguishable from those with *Rapgef2*-cKO mice on the mixed background of ICR and C57BL/6J (Bilasy et al., 2009).

On the other hand, the total numbers of Ctip2^+^ neurons and Cux1^+^ neurons showed a substantial increase in the *Rapgef2/6*-dKO cortices, while that of Cux1^+^ neurons only showed an increase in the *Rapgef2*-cKO cortices (Fig. 1*D*), hinting that an expansion of the neural progenitor pools for the affected neurons might have occurred in *Rapgef2*-cKO and *Rapgef2/6*-dKO embryos.

### Effects of *Rapgef*2-cKO and *Rapgef2/6*-dKO on distribution of neural progenitors in the developing cerebral cortex

**Figure 2.**
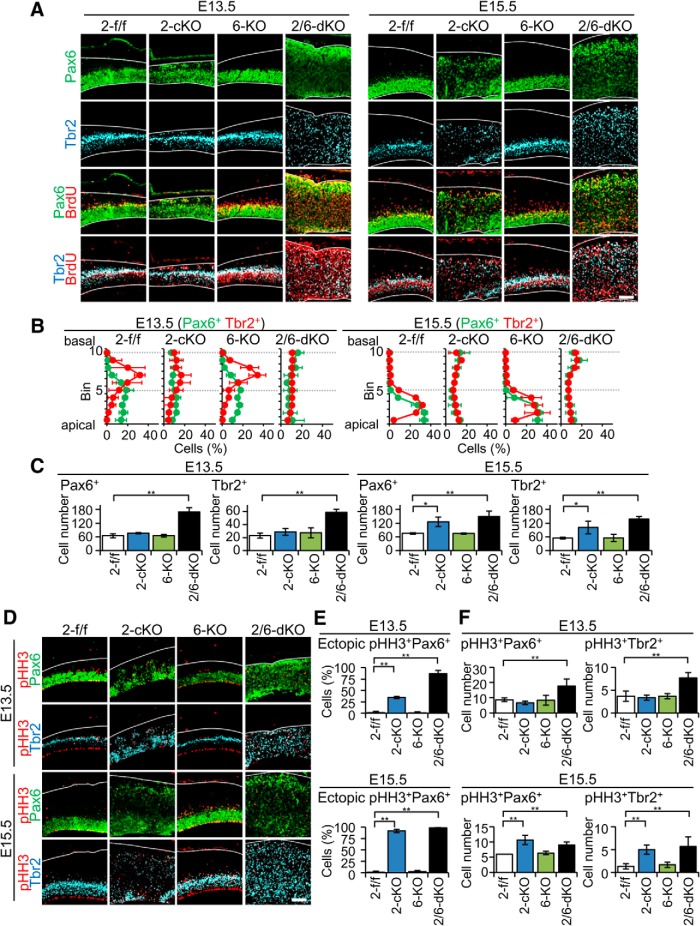
Effects of the genotypes of Rapgef2 and Rapgef6 on the distribution of neural progenitor cells. ***A***, Immunohistological detection of Pax6^+^ cells, Tbr2^+^ cells, and BrdU-labeled S-phase cells. Coronal sections of the brains of *Rapgef2*-f/f (*2-f/f*), *Rapgef2*-cKO (*2-cKO*), *Rapgef6*-KO (*6-KO*), and *Rapgef2/6*-dKO (*2/6-dKO*) embryos at E13.5 and E15.5, which had been subjected to BrdU labeling, were subjected to immunostaining for Pax6 (green), Tbr2 (cyan), and BrdU (red). Pial and apical surfaces of the cortices are indicated by white lines. The images are representative of four biological replicates of each group. Scale bars, 100 µm. ***B***, ***C***, Distribution of Pax6^+^ cells and Tbr2^+^ cells in the cerebral cortices. Brain sections prepared and stained as in ***A*** were subjected to the quantification of Pax6^+^ cells (green lines) and Tbr2^+^ cells (red lines) residing in each bin, as described in Materials and Methods (***B***). Data are expressed as mean ± SD values derived from at least four biological replicates. The progenitors contained in a total of 10 bins (100 μm in width) were counted as described in Materials and Methods (***C***). Data are expressed as the mean ± SD values derived from four mice of each genotype. Student’s *t* test: *p < 0.05, **p < 0.01. ***D***, Immunohistological detection of Pax6^+^ cells, Tbr2^+^ cells, and pHH3^+^ metaphase cells. Brain sections prepared as in ***A*** were subjected to immunostaining for Pax6 (green), Tbr2 (cyan), and pHH3 (red). Pial surfaces are indicated by white lines. The images are representative of four biological replicates of each group. Scale bars, 100 µm. ***E***, ***F***, Ectopic localization of Pax6^+^ and Tbr2^+^ metaphase cells. The percentages of the ectopically localized somata of pHH3^+^/Pax6^+^ cells in the total pHH3^+^/Pax6^+^ cells contained in the 10 bins (100 μm in width) were determined using the immunostained sections described in ***D*** (***E***). Also, pHH3^+^/Pax6^+^ cells and pHH3^+^/Tbr2^+^ cells in a total of 10 bins (100 μm in width) were counted. Data are expressed as the mean ± SD values derived from four mice of each genotype. Student’s *t* test: **p < 0.01.

To gain an insight into the mechanism for the ECM formation, we next examined the courses of brain development of *Rapgef2*-cKO and *Rapgef2/6*-dKO embryos at E13.5 and E15.5, when early-born neurons and late-born neurons, respectively, were predominantly generated (Greig et al., 2013). Differentiation from RGCs to neurons could be traced by sequential alterations in the expression of the transcription factors, as follows: Pax6 in RGCs; and Tbr2 in IPCs (Englund et al., 2005). At E13.5 and E15.5, Pax6^+^ RGCs and Tbr2^+^ IPCs were exclusively located in the VZ (bins 1-5 at E13.5, and bins 1-3 at E15.5) and the SVZ (bins 6-8 at E13.5, and bins 2-4 at E15.5), respectively, in the *Rapgef2*-f/f and *Rapgef6*-KO cortices (Fig. 2*A*,*B*). On the other hand, at E13.5 in the *Rapgef2*-cKO cortices, minor but significant populations of Pax6^+^ cells and Tbr2^+^ cells were also located at the basal side (bins 9 and 10). Strikingly, in the *Rapgef2*/*6*-dKO cortices, both Pax6^+^ cells and Tbr2^+^ cells were scattered almost evenly over their entire thickness at both E13.5 and E15.5. Moreover, at E15.5, the cortex-wide scattering of both Pax6^+^ RGCs and Tbr2^+^ IPCs also became evident in the *Rapgef2*-cKO cortices (Fig. 2*A*,*B*). Similar to the observation with neurons at P90, the total numbers of Pax6^+^ RGCs and Tbr2^+^ IPCs were substantially increased at both E13.5 and E15.5 in the *Rapgef2/6*-dKO cortices, and at E15.5 only in the *Rapgef2*-cKO cortices (Fig. 2*C*). Consistent with this, a substantial increase in the cortical thickness was observed in *Rapgef2/6*-dKO brains at both E13.5 and E15.5 and in the *Rapgef2*-cKO brains at E15.5 only (Fig. 2*A*).

Next, we used BrdU labeling to visualize cells in the S phase of the cell cycle (Fig. 2*A*). At E13.5, almost all BrdU^+^ cells were also positive for either Pax6 or Tbr2 and were located at the basal side of the VZ and the apical side of the SVZ, respectively, in the *Rapgef2*-f/f and *Rapgef6*-KO cortices. On the other hand, in the *Rapgef2*-cKO cortices at E13.5, minor but significant populations of BrdU^+^/Pax6^+^ cells and BrdU^+^/Tbr2^+^ cells were located ectopically at the basal side (bins 9 and 10). At E15.5, both BrdU^+^/Pax6^+^ cells and BrdU^+^/Tbr2^+^ cells were evenly scattered over the entire cortex. Notably, the *Rapgef2/6*-dKO cortices showed similar cortex-wide scattering of both BrdU^+^/Pax6^+^ cells and BrdU^+^/Tbr2^+^ cells by E13.5. Furthermore, the *Rapgef2*-cKO and *Rapgef2/6*-dKO cortices showed an essentially similar pattern of the ectopic localization of metaphase cells, which were visualized by immunostaining for phospho-histone-H3 (Ser10; pHH3; Fig. 2*D*). Notably, in the *Rapgef2/6*-dKO cortices at both E13.5 and E15.5, and the *Rapgef2*-cKO cortices at E15.5 only, pHH3^+^/Pax6^+^ and pHH3^+^/Tbr2^+^ cells lost their normal localizations at the apical sides of the VZ and the SVZ, respectively, and were almost evenly scattered over their entire thickness. The percentages of pHH3^+^/Pax6^+^ cells located in close proximity to the apical surface were markedly reduced in the *Rapgef2/6*-dKO cortices at both E13.5 and E15.5, and the *Rapgef2*-cKO embryos at E15.5 only, compared with almost 100% in the *Rapgef2*-f/f and *Rapgef6*-KO cortices at both E13.5 and E15.5 (Fig. 2*E*). As observed with Pax6^+^ and Tbr2^+^ cells, the total numbers of pHH3^+^/Pax6^+^ and pHH3^+^/Tbr2^+^ cells were substantially increased at both E13.5 and E15.5 in the *Rapgef2/6*-dKO cortices and at E15.5 only in the *Rapgef2*-cKO cortex (Fig. 2*F*). Similar ectopic localization of metaphase RGCs had been observed in mice deficient in AJ proteins (Lien et al., 2006; Kadowaki et al., 2007; Gil-Sanz et al., 2014; Yamamoto et al., 2015).

### Effects of *Rapgef2*-cKO and *Rapgef2/6*-dKO on the apical surface structure in the developing cerebral cortex

**Figure 3.**
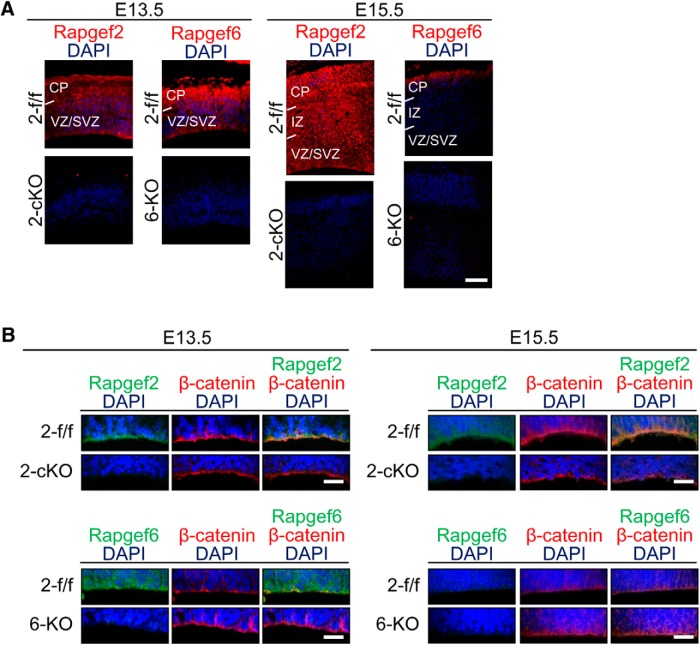
Expression of Rapgef2 and Rapgef6 in the E13.5 and E15.5 brains. ***A***, Immunohistological detection of Rapgef2 and Rapgef6. Coronal sections of the brains of *Rapgef2*-f/f (*2-f/f*), *Rapgef2*-cKO (*2-cKO*), and *Rapgef6*-KO (*6-KO*) embryos at E13.5 and E15.5 were subjected to immunostaining for Rapgef2 or Rapgef6 (red) and 4',6-diamidino-2-phenylindole (DAPI; blue). Scale bars, 100 µm. ***B***, Preferential expression of Rapgef2 and Rapgef6 on the apical surfaces. Coronal sections of the brains of the E13.5 and E15.5 embryos with the indicated genotypes were subjected to immunostaining for Rapgef2 or Rapgef6 (green), β-catenin (red), and DAPI (blue). The images are representative of four biological replicates of each group. Scale bars, 20 µm.

**Figure 4.**
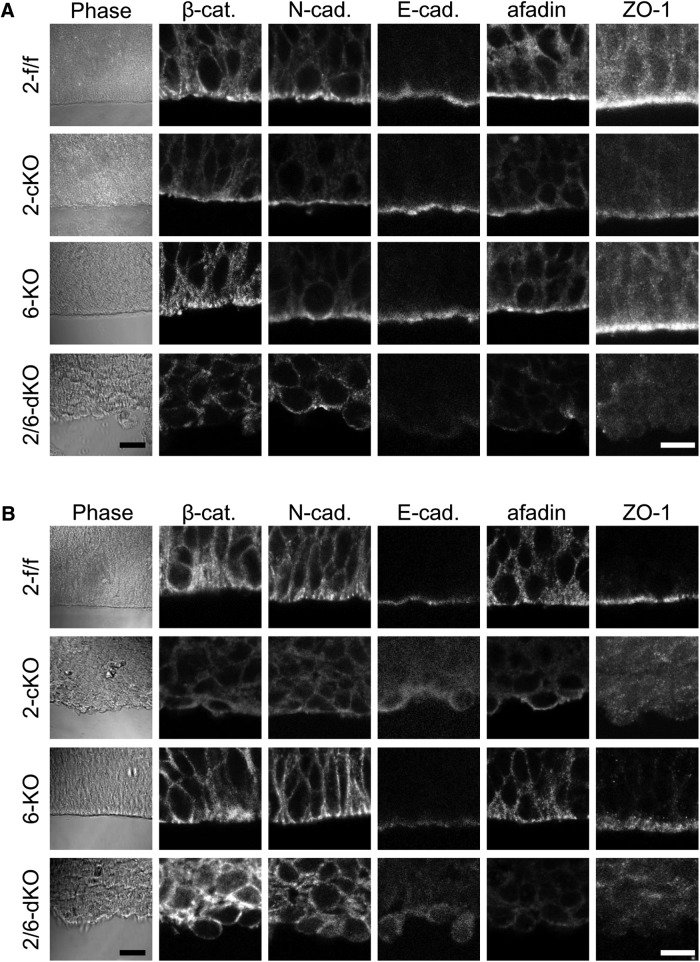
Effects of the genotypes of Rapgef2 and Rapgef6 on the apical surface structures. ***A***, ***B***, Immunohistological detection of proteins constituting the AJs. ***A***, ***B***, Coronal sections of the brains of *Rapgef2*-f/f (*2-f/f*), *Rapgef2*-cKO (*2-cKO*), *Rapgef6*-KO (*6-KO*), and *Rapgef2/6*-dKO (*2/6-dKO*) embryos at E13.5 (***A***) and E15.5 (***B***) were subjected to immunostaining for β-catenin (β-cat.), N-cadherin (N-cad.), E-cadherin (E-cad.), afadin, and ZO-1, as indicated. The leftmost panels are phase contrast images with a lower magnification. The images are representative of four biological replicates of each group. Scale bars: leftmost panels, 100 µm; other panels, 10 µm.

We next analyzed the expression pattern of Rapgef2 and Rapgef6 in the developing cerebral cortex. Immunostaining of the E13.5 brain sections showed that both Rapgef2 and Rapgef6 were expressed throughout the cortices with a higher extent of expression at the apical surface (Fig. 3*A*). However, at E15.5 Rapgef6 disappeared from the VZ/SVZ and IZ, and the apical part of the CP, whereas Rapgef2 retained an expression pattern similar to that observed at E13.5. The specificities of the immunostaining were verified by the disappearance of the immunoreactive signals concomitant with the knockout of the corresponding genes. The apical surface structure lining the ventricles is composed of an assembly of the end feet of the apical RG fibers, which are linked together by an array of the AJs (Götz and Huttner, 2005). Both Rapgef2 and Rapgef6 at E13.5, and Rapgef2 only at E15.5, were concentrated at the AJs on the apical surface as shown by their colocalization with β-catenin (Fig. 3*B*). Considering that the constituents of the AJs, such as β-catenin, N-cadherin, E-cadherin, afadin, and ZO-1, play critical roles in formation of the apical surface structure and that ablation of their individual functions led to ECM formation similar to that observed in the *Rapgef2*-cKO brain (Machon et al., 2003; Junghans et al., 2005; Lien et al., 2006; Kadowaki et al., 2007; Gil-Sanz et al., 2014; Schmid et al., 2014; Yamamoto et al., 2015), we examined the apical surface morphology of the *Rapgef2*-cKO and *Rapgef2/6*-dKO cortices (Fig. 4). Strikingly, in the *Rapgef2/6*-dKO cortices at both E13.5 and E15.5, their apical surfaces became very irregular and heavily disintegrated so that a significant population of cells was apparently exfoliated from the surfaces into the ventricle (Fig. 4*A*,*B*, leftmost panels). The *Rapgef2*-cKO cortices showed less severe morphological abnormalities; the apical surfaces looked almost intact at E13.5 but assumed irregular morphology at E15.5. The formation of the AJs lining the apical surface was further examined by immunostaining for β-catenin, N-cadherin, E-cadherin, afadin, and ZO-1, all of which were concentrated there in the *Rapgef2*-f/f and *Rapgef6*-KO cortices at E13.5 and E15.5 (Fig. 4*A*,*B*). Strikingly, in the *Rapgef2/6*-dKO cortices, a dense array of the apical surface AJs became almost nonexistent at both E13.5 and E15.5. Again, the *Rapgef2*-cKO cortices showed less severe abnormalities; the disappearance of the AJs was not clearly recognizable until E15.5.

### Effects of *Rapgef2*-cKO and *Rapgef2/6*-dKO on the RG fiber organization

**Figure 5.**
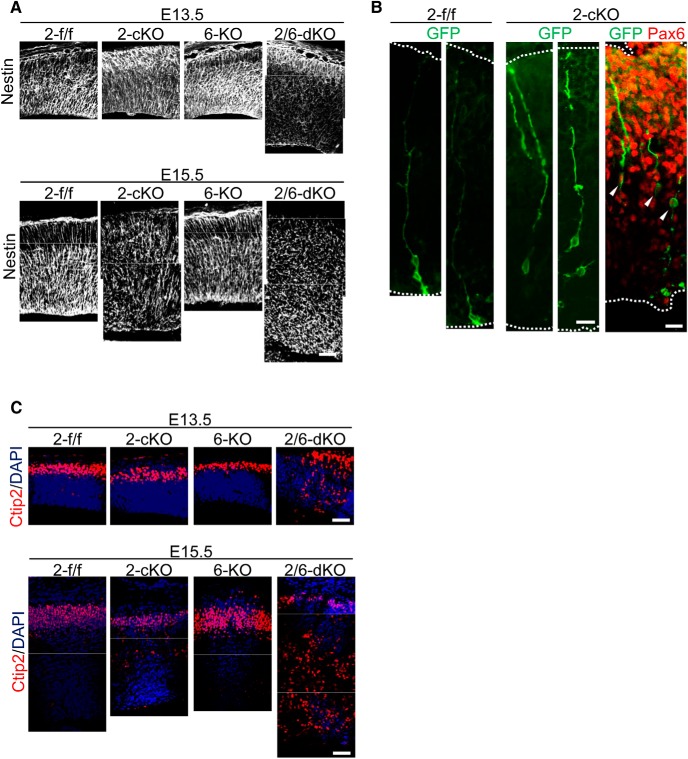
Effects the genotypes of Rapgef2 and Rapgef6 on RG fiber organization and neuronal migration. ***A***, Morphology of RG fibers. Coronal sections of the brains of *Rapgef2*-f/f (2-f/f), *Rapgef2*-cKO (2-cKO), *Rapgef6*-KO (6-KO), and *Rapgef2/6*-dKO (2/6-dKO) embryos at E13.5 and E15.5 were subjected to immunostaining for nestin. The images are representative of three biological replicates of each group. Scale bars, 50 µm. ***B***, Morphology of ectopically located GFP-labeled cells. RGCs lining the apical surface of the 2-f/f and 2-cKO cortices were labeled with GFP by IUE of pCAG-EGFP-F at E14.5, and the morphology of their progenies was analyzed at E15.5 by immunohistological staining for GFP (green) and Pax6 (red). GFP^+^/Pax6^+^ RGCs are indicated by arrowheads. The apical and pial surfaces of the telencephalon were indicated by white broken lines. The images are representative of four biological replicates of each group. Scale bars: 20 µm ***C***, Localization of Ctip2^+^ neurons. Coronal sections of the brains of the E13.5 and E15.5 embryos with the indicated genotypes were subjected to immunostaining for Ctip2 (red) and DAPI (blue). The images are representative of three biological replicates of each group. Scale bars, 50 µm.

Regarding the past studies showing that AJ disruption impaired the scaffolding of RGCs on the apical surface and caused a disturbance of the RG fiber organization (Kadowaki et al., 2007; Yamamoto et al., 2015), we examined RG fiber morphology in the *Rapgef2*-cKO and *Rapgef2/6*-dKO cortices by immunostaining for nestin. In the *Rapgef2*-f/f and *Rapgef6*-KO cortices, nestin^+^ RG fibers showed regular radial alignment spanning their entire thickness and reaching the pial surface (Fig. 5*A*). Notably, in the *Rapgef2*/*6*-dKO cortices at both E13.5 and E15.5, the RG fiber system was heavily disorganized; RG fibers were randomly oriented throughout their entire thickness. Moreover, the RG fibers seemed to be broken down into short pieces at E15.5, which might reflect the dispersion of RGCs throughout the cortices. On the other hand, in the *Rapgef2*-cKO cortices, the disorganization of RG fibers was not clearly recognizable until E15.5 when RG fibers showed disoriented and irregular arrangements more prominent in the lower zones including the SVZ/VZ and IZ than in the upper zones including the CP. The time course of the RG fiber disorganization apparently correlated with that of the apical surface AJ disruption. Next, IUE-mediated transduction of pCAG-EGFP-F, expressing plasma membrane-localized EGFP, into E14.5 *Rapgef2*-cKO embryos was used to more clearly observe the RG fiber morphology in the E15.5 cortices (Fig. 5*B*). While the basal fibers of the ectopically located Pax6^+^ RGCs looked almost intact reaching the pial surface, the apical fibers became shorter or almost nonexistent and seemed to be disconnected from the apical surface.

At the same time, we examined the migration of Ctip2^+^ neurons, which is dependent on the RG fiber-guided locomotion. In the *Rapgef2*-f/f and *Rapgef6*-KO cortices, Ctip2^+^ neurons were almost exclusively located in the CP at E13.5 and E15.5 (Fig. 5*C*). In sharp contrast, in the *Rapgef2/6*-dKO cortices, most of Ctip2^+^ neuron failed to reach the CP and were located in the lower zones at E15.5. The sign of this migration delay was already evident at E13.5. In the *Rapgef2*-cKO cortices, the location of Ctip2^+^ neurons looked normal at E13.5. However, a small but significant proportion of Ctip2^+^ neurons were located in the lower zones at E15.5. These results suggested the existence of a certain correlation between the extents of RG fiber disorganization and the neuronal migration disorder. Also, the ectopic accumulation of neurons observed at E15.5 was likely to be a predecessor of the ECM formed in the adult *Rapgef2*-cKO and *Rapgef2*/*6*-dKO cortices.

### Cell-autonomous function of Rapgef2 in neural progenitors and role of its Rap1-GEF activity

**Figure 6.**
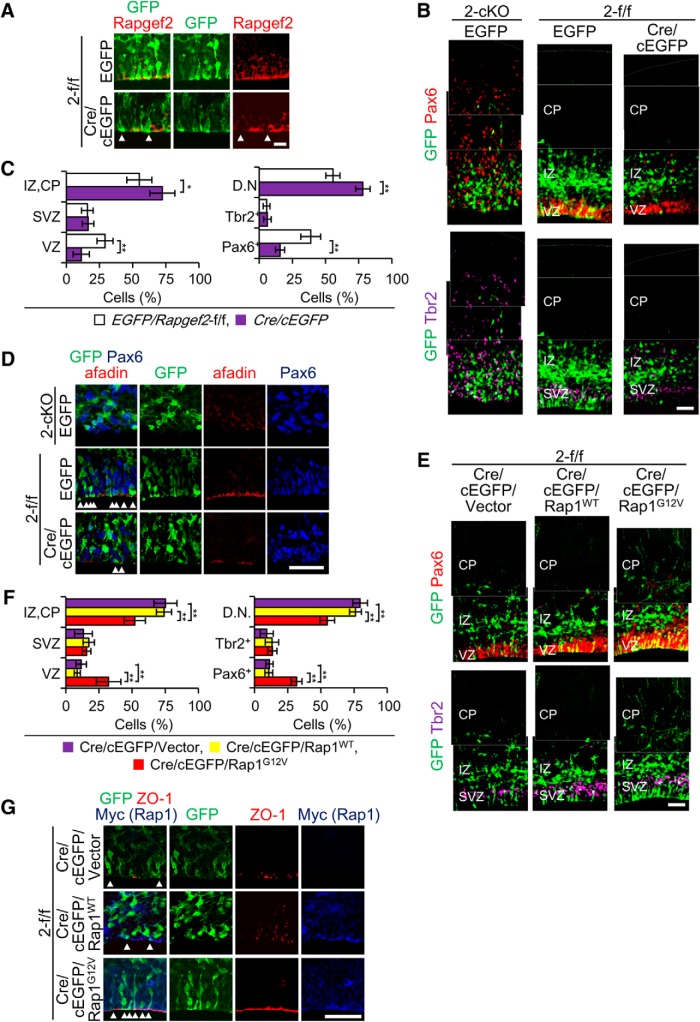
Phenotypes of Cre-mediated Rapgef2 knockout and compensatory effects of artificial Rap1 expression. ***A***, Efficient knockout of *Rapgef2* by introduction of Cre recombinase. *Rapgef2*-f/f (2-f/f) embryos at E14.5 were subjected to IUE-mediated transduction of pCAG-NLS-Cre and pCAG-FloxP-EGFP-N1 (Cre/cEGFP). As controls, *Rapgef2-*cKO (2-cKO), and *Rapgef2*-f/f embryos at E14.5 were subjected to IUE-mediated transduction of CAG=EGFP (EGFP). Coronal sections of the brains of *Cre/cEGFP* and *EGFP/Rapgef2*-f/f embryos at E16.5 were subjected to immunostaining for GFP (green) and Rapgef2 (red), as indicated. GFP^+^ cells apparently lacking Rapgef2 expression are indicated by arrowheads. The images are representative of four biological replicates of each group. Scale bars, 20 µm. ***B***, ***C***, Zonal distribution of GFP-labeled cells and their neural progenitor markers. The sections prepared as in ***A*** were subjected to immunostaining for GFP (green), Pax6 (red), and Tbr2 (purple; ***B***). The images are representative of four biological replicates of each group. Scale bars, 50 µm. The percentages of GFP^+^ cells located in the VZ, SVZ, and IZ/CP (left), and those displaying Pax6^−^/Tbr2^−^ (D.N.), Pax6^+^, and Tbr2^+^ markers (right) are shown as mean ± SD values derived from four each of *EGFP/Rapgef2-ff* (white bars) and *Cre/cEGFP* (purple bars) embryos (***C***). Student’s *t* test: *p < 0.05, **p < 0.01. ***D***, Disruption of the apical surface structures by Cre-mediated *Rapgef2* knockout. The sections prepared as in ***A*** were subjected to immunostaining for GFP (green), afadin (red), and Pax6 (blue), as indicated. The images are representative of four biological replicates of each group. Arrowheads indicate the end feet of the apical fibers of GFP-labeled RGCs on the apical surface. Scale bars, 50 µm. ***E***, ***F***, Effects of the overexpression of constitutively active Rap1 on zonal distribution of GFP-labeled cells and their neural progenitor markers. pCAG-Myc-Rap1^WT^ (Rap1^WT^), pCAG-Myc-Rap1^G12V^ (Rap1^G12V^), or pCAG-Myc (Vector) were cotransduced by IUE with pCAG-NLS-Cre and pCAG-FloxP-EGFP-N1 into *Rapgef2*-f/f (2-f/f) embryos at E14.5. Coronal sections of the brains at E16.5 were subjected to immunostaining for GFP (green), Pax6 (red), and Tbr2 (purple), as indicated (***E***). The images are representative of three biological replicates of each group. Scale bars, 50 µm. The percentages of GFP^+^ cells located in the VZ, SVZ, and IZ/CP (left) and those displaying Pax6^−^/Tbr2^−^ (D.N.), Pax6^+^, and Tbr2^+^ markers (right) are shown as mean ± SD values derived from three each of mice cotransduced with pCAG-Myc (purple bars), pCAG-Myc-Rap1^WT^ (yellow bars), and pCAG-Myc-Rap1^G12V^ (red bars; ***F***). Student’s *t* test: **p < 0.01. ***G***, Effects of Rap1 overexpression on the apical surface structures of Cre-mediated *Rapgef2* knockout. The sections prepared as in ***E*** were subjected to immunostaining for GFP (green), ZO-1 (red), and Myc (blue), as indicated. The images are representative of three biological replicates of each group. Arrowheads indicate the end feet of the apical fibers of GFP-labeled cells on the apical surface. Scale bars, 50 µm.

To examine the cell-autonomous function of Rapgef2, we introduced *Rapgef2* knockout into RGCs lining the apical surface by IUE-mediated transduction of pCAG-NLS-Cre, expressing Cre recombinase, into *Rapgef2*-f/f embryos at E14.5. Cells harboring the Cre-expression vector were marked by GFP through cotransduction of pCAG-FloxP-EGFP-N1, which conditionally expresses EGFP in cells expressing Cre recombinase (Shitamukai et al., 2011). Hereafter, the resulting embryos are called *Cre/cEGFP*. As control, RGCs were labeled with GFP by IUE-mediated transduction of pCAG=EGFP into *Rapgef2*-f/f and *Rapgef2*-cKO embryos at E14.5 to trace their fate, and the resulting embryos are called *EGFP/Rapgef2*-f/f and *EGFP/Rapgef2*-cKO, respectively. When examined at E16.5, Rapgef2 expression was successfully eliminated in GFP^+^ cells in the *Cre/cEGFP* cortices (Fig. 6*A*). In *EGFP/Rapgef2*-cKO embryos at E16.5, Pax6^+^/GFP^+^ cells, exclusively located in the VZ in the case of *EGFP/Rapgef2*-f/f embryos, almost completely disappeared from the VZ and were mainly located in the IZ and CP, as observed with Pax6^+^ cells as a whole (Figs. 2*A*,*B*, 6*B*). Likewise, the majority of Tbr2^+^/GFP^+^ cells, predominantly located in the SVZ in *EGFP/Rapgef2*-f/f embryos, showed ectopic localization in the IZ and CP in *EGFP/Rapgef2*-cKO embryos, as observed with Tbr2^+^ cells as a whole. Likewise, in *Cre/cEGFP* embryos at E16.5, the proportion of Pax6^+^/GFP^+^ cells was also markedly decreased in the VZ, which was accompanied by an increase in the proportion of GFP^+^ cells located in the IZ (Fig. 6*B*,*C*). However, the GFP^+^ cells in the IZ were totally negative for Pax6 or Tbr2, indicating that they lost the progenitor property. The distribution of Tbr2^+^/GFP^+^ cells appeared not significantly affected (Fig. 6*B*,*C*). The apical surface of the *EGFP/Rapgef2*-cKO cortices showed very irregular cell arrangements and disappearance of the apical fiber end feet of Pax6^+^/GFP^+^ cells, which was accompanied by the loss of the apical fibers in Pax6^+^/GFP^+^ cells (Fig. 6*D*). Moreover, immunostaining for Pax6 and afadin showed that the AJs on the apical surface became almost nonexistent. These results were consistent with the observation with the *Rapgef2*-cKO cortices at E15.5 and the *Rapgef2/6*-dKO cortices at E13.5 and E15.5 (Fig. 4). In the *Cre/cEGFP* cortices, the numbers of GFP^+^ apical fibers and their end feet on the apical surface were also substantially reduced, and the apical surface AJs were compromised, as shown by immunostaining for afadin (Fig. 6*D*). Together, these results indicated that Rapgef2 possesses a cell-autonomous function in maintaining the apical surface AJ structures and preventing earlier detachment of RGCs from the apical surface. However, a non-cell-autonomous effect of the *Rapgef2* knockout seems to be responsible for preservation of the progenitor properties in RGCs and IPCs detached from the apical surface observed in the *Rapgef2*-cKO cortices.

To clarify whether the function of Rapgef2 in RGCs was governed by their GEF activity toward Rap1, we examined the effects of artificial expression of Rap1^G12V^, the constitutively active Rap1 mutant that had its GTPase activity severely impaired and did not rely on Rap1-GEFs for its activation. Rap1^WT^, wild-type Rap1, whose activity was dependent on Rap1-GEFs, was used as a control. To this end, pCAG-Myc-Rap1^G12V^, pCAG-Myc-Rap1^WT^, or pCAG-Myc empty vector was transduced by IUE into *Rapgef2*-f/f embryos in combination with both pCAG-NLS-Cre and pCAG-FloxP-EGFP-N1 at E14.5. When observed at E16.5, the cotransduction of pCAG-Myc-Rap1^G12V^, but not pCAG-Myc-Rap1^WT^ or pCAG-Myc, successfully restored not only the proportion of Pax6^+^/GFP^+^ cells in the VZ (Fig. 6*E*,*F*), but also the numbers of the apical fibers and their end feet and the density of the apical surface AJs (Fig. 6*G*) to the extent comparable with that of the *EGFP/Rapgef2*-f/f cortex (Fig. 6*B–D*). These results indicated that the Rap1-GEF activity of Rapgef2 was responsible for its function in maintaining the apical surface AJ structures and preventing earlier detachment of RGCs from the apical surface.

### Cell-autonomous and non-cell-autonomous functions of Rapgef2 on neuronal migration

**Figure 7.**
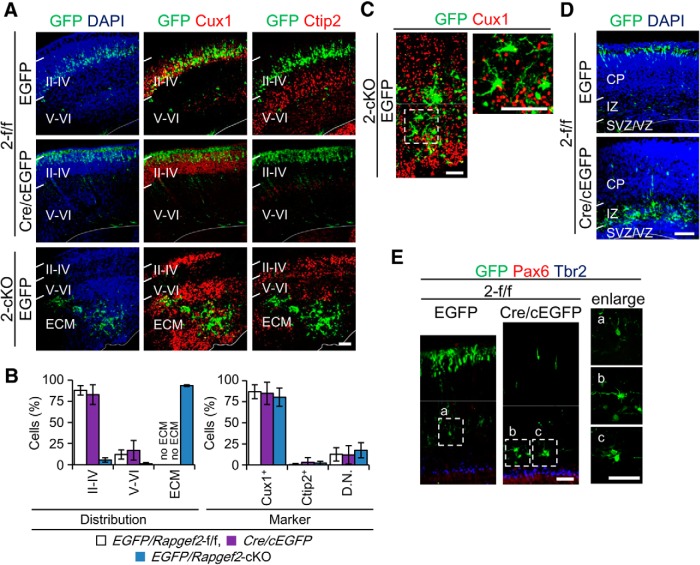
Effects of *Rapgef2* knockout on the morphology and migration of neurons. ***A***, ***B***, Effects of *Rapgef2* knockout on neuronal migration observed at P7. *Rapgef2*-f/f (2-f/f) embryos at E14.5 were subjected to IUE-mediated transduction of pCAG-NLS-Cre and pCAG-FloxP-EGFP-N1 (Cre/cEGFP). As controls, *Rapgef2-*cKO (2-cKO) and *Rapgef2*-f/f embryos at E14.5 were subjected to IUE-mediated transduction of CAG=EGFP (EGFP). Coronal sections of the brains at P7 were subjected to immunostaining for GFP (green), Cux1 (red), Ctip2 (red), and DAPI (blue), as indicated (***A***). Due to the nuclear localizing nature of EGFP, double staining with Cux1 or Ctip2 is recognizable only in the nuclei. Images shown are representative of four biological replicates of each group. Layers II–IV and V–VI and the ECM are indicated. The apical surfaces of the cortices are indicated by white lines. Scale bars, 100 µm. ***B***, The percentages of GFP^+^ cells located in thelayers II–IV, V and VI and the ECM (left), and those displaying Cux1^+^, Ctip2^+^, and Cux1^−^/Ctip2^−^ (D.N.) markers (right) are shown as mean ± SD values derived from eight *EGFP/Rapgef2*-f/f embryos (white bars), four *EGFP/Rapgef2*-cKO embryos (blue bars), and four *Cre/EGFP* embryos (purple bars). ***C***, Morphology of GFP^+^/Cux1^+^ neurons located in the ECM of the *Rapgef2*-cKO embryo at P7. A brain section of the *EGFP/Rapgef2*-cKO embryo prepared as in ***A*** was subjected to immunostaining for GFP (green) and Cux1 (red). Right, A magnified image of the area surrounded by a broken line. Scale bars, 50 µm. ***D***, Effects of Cre-mediated *Rapgef2* knockdown on neuronal migration observed at E18.5. Coronal sections of the brains were prepared at E18.5 from the *Cre/EGFP* and *EGFP/Rapgef2*-f/f embryos generated by IUE at E14.5 as described in ***A*** and subjected to immunostaining for GFP (green) and DAPI (blue). The images are representative of four biological replicates of each group. The apical surfaces of the cortices are indicated by white lines. Scale bars, 100 µm. ***E***, Morphology of GFP^+^ cells located in the IZ at E18.5. The brain sections of the E18.5 *Cre/EGFP* and *EGFP/Rapgef2*-f/f embryos IUE were subjected to immunostaining for GFP (green), Pax6 (red), and Tbr2 (blue). ***E***, Left panels (***a****–****c***) are magnified images of the corresponding areas surrounded by broken lines. Scale bars, 50 µm.

Rap1 plays crucial roles in neuronal migration, particularly in the multipolar migration, the multipolar–bipolar transition, and the terminal translocation (Voss et al., 2008; Franco et al., 2011; Jossin and Cooper, 2011; Sekine et al., 2012; Ye et al., 2014). To clarify the role of Rapgef2 in neuronal migration, the morphology and location of neurons labeled with GFP by IUE at E14.5 were examined at P7 in *EGFP/Rapgef2*-f/f and *EGFP/Rapgef2*-cKO mice (Fig. 7*A*). In the *EGFP/Rapgef2*-f/f cortices, GFP^+^ cells were late-born neurons positive for Cux1 and almost exclusively located in layers II–IV (Fig. 7*A*,*B*). In contrast, consistent with the ECM formation predominantly consisting of late-born neurons, almost all of the GFP^+^ cells, which were also positive for Cux1, were located in the ECM in the *EGFP/Rapgef2*-cKO cortices. Notably, about a half of them assumed multipolar morphology (Fig. 7*C*), suggesting that an impairment of the multipolar–bipolar transition caused the defect in neuronal migration. To examine the cell-autonomous effect of the *Rapgef2* knockout, we observed the morphology and location of neurons at P7 in *Cre/cEGFP* mice. Notably, almost all of Cux1^+^/GFP^+^ neurons successfully reached layers II–IV (Fig. 7*A*,*B*). Closer inspection revealed that they were mainly located in the top section, probably corresponding to layer II–III, rather than the bottom section, probably corresponding to layer IV observed in the *EGFP/Rapgef2*-f/f cortices, suggesting delayed migration of Rapgef2-deficient neurons in the *Cre/cEGFP* cortices (Fig. 7*A*). When the time course of migration was examined at E18.5, the proportion of GFP^+^ cells in the IZ was markedly increased in the *Cre/cEGFP* cortices (Fig. 7*D*). At the same time, while 11.7 ± 3.4% of GFP^+^ cells assumed multipolar morphology in the *EGFP/Rapgef2*-f/f cortices, 52.1 ± 1.3% of GFP^+^ cells exhibited multipolar morphology and remained in the IZ in the *Cre/cEGFP* cortices (Fig. 7*E*), indicating the existence of a multipolar–bipolar transition defect, which was similar to that observed in mice with the Rap1GAP-mediated inactivation of Rap1 or the shRNA-mediated knockdown of Rapgef2 (Jossin and Cooper, 2011; Ye et al., 2014). These results suggested that the loss of the Rapgef2 function caused transient defects in the migration and multipolar–bipolar transition of postmitotic neurons by a cell-autonomous mechanism. The corresponding defects in the *Rapgef2*-cKO cortices, which last to adulthood and lead to ECM formation, seemed to be caused by a non-cell-autonomous effect.

**Figure 8.**
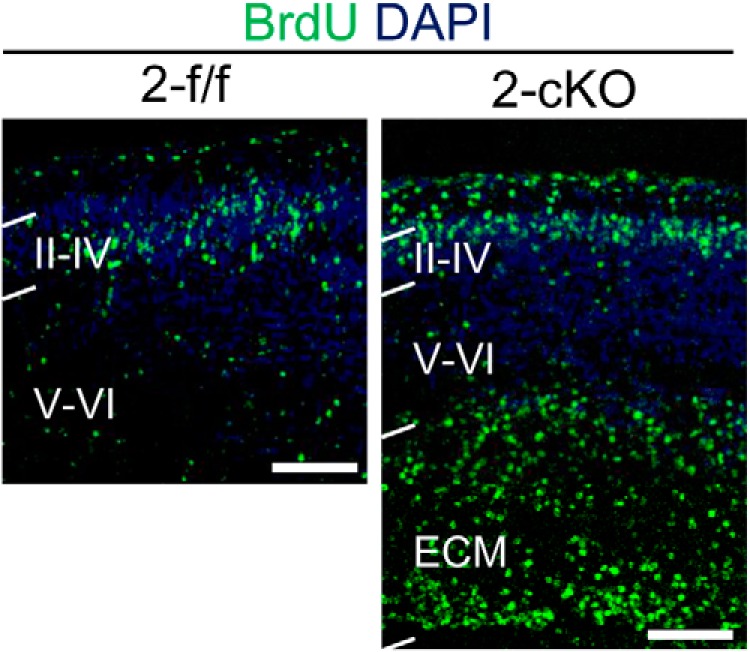
Distribution of neurons derived from neural progenitors labeled by BrdU at E15.5. Neural progenitors in E15.5 *Rapgef2*-f/f (2-f/f) and *Rapgef2*-cKO (2-cKO) embryos were labeled by BrdU, and neurons derived from them were detected by immunohistological staining for BrdU at P0. Nuclei were stained with DAPI. Layers II–IV and V–VI, and the ECM are indicated. Images shown are representative of four biological replicates of each group. Scale bars, 100 μm.

The results obtained with the *EGFP/Rapgef2*-cKO cortices also revealed that almost all of GFP^+^ neurons failed to migrate and were localized in the IZ at P7 (Fig. 7*A*,*B*), indicating that RGCs lining the apical surface, which could be labeled with GFP by IUE, did not constitute the progenitor pool for neurons constituting the six-layered neocortex. To examine the role of ectopically located progenitors in generating neurons in the neocortex, we used the BrdU incorporation method, which was capable of labeling neural progenitors regardless of their localization. When BrdU was administered to pregnant mice with *Rapgef2*-f/f and *Rapgef2*-cKO embryos at E15.5 to label late-born neurons, BrdU^+^ cells were found to be located in both layers II–IV and the ECM at P0 in the *Rapgef2*-cKO cortices, while they were predominantly located in layers II–IV in the *Rapgef2*-f/f cortices (Fig. 8). These results implied that, in the E14.5 *Rapgef2*-cKO cerebral cortices, ectopically located RGCs and IPCs, not RGCs located in the VZ, mainly contributed to the formation of the six-layered neocortex.

## Discussion

Rap1 has been shown to play crucial roles in the following various processes of cortical development: the preplate splitting, the multipolar–bipolar transition prerequisite for the RGC-independent multipolar migration, and the RGC-independent terminal translocation, but not the RG fiber-dependent locomotion of postmitotic neurons (Voss et al., 2008; Franco et al., 2011; Jossin and Cooper, 2011; Sekine et al., 2012; Ye et al., 2014). This is based on the observation of mouse embryonic brains with shRNA-mediated knockdown of Rap1 or Rap1-GEFs, such as Rapgef1/C3G and Rapgef2, and IUE-mediated overexpression of a dominant-negative Rap1 mutant or a Rap1-GAP as well as those with a hypomorphic mutation of Rapgef1/C3G. The Reelin-Reelin receptor Dab1-Crk/CrkL-Rapgef1/C3G-Rap1 pathway is involved in all of the Rap1-dependent processes described above, although molecules downstream of Rap1 seem to be different, as follows: N-cadherin for the multipolar migration and multipolar–bipolar transition, and integrin α5β1 and N-cadherin for the terminal translocation (Voss et al., 2008; Franco et al., 2011; Jossin and Cooper, 2011; Sekine et al., 2012). However, it was recently reported that Rapgef2, not Rapgef1/C3G, specifically governs the multipolar–bipolar transition via Rap1-N-cadherin signaling and that its activity is regulated through phosphorylation by Cdk5 (Ye et al., 2014). In addition, Rap1 seems to be involved in other neurodevelopmental processes such as dendrite development (Chen et al., 2005; Srivastava et al., 2012) and spine morphogenesis (Xie et al., 2005).

In this study, we demonstrated that Rapgef6 has a cooperative function with Rapgef2 in cortical development by showing that additional knockout of *Rapgef6* in *Rapgef2*-cKO mice resulted in marked enlargement of the ECM lacking the layer structure at P90, while knockout of *Rapgef6* alone did not exhibit any discernible effects (Fig. 1). While the *Rapgef2*-cKO ECM was composed of late-born neurons only, the *Rapgef2/6*-dKO ECM was composed of both early-born and late-born neurons, suggesting the earlier occurrence of developmental defects in the *Rapgef2/6*-dKO brains. These observations prompted us to study the effects of *Rapgef2*-cKO and *Rapgef2/6*-dKO on the differentiation and migration of neural progenitors and neurons during embryonic brain development. To our surprise, we found that Pax6^+^ RGCs and Tbr2^+^ IPCs were scattered over the entire cerebral cortex, and that their total numbers showed a substantial increase in *Rapgef2*-cKO embryos at E15.5 and *Rapgef2/6*-dKO embryos at E13.5 and E15.5 (Fig. 2). Concurrently, both mitotic cells and S-phase cells, which were either Pax6^+^ or Tbr2^+^, also showed similar cortex-wide scattering and an increase in their numbers, indicating that the ectopically located Pax6^+^ cells and Tbr2^+^ cells were proliferative and that an expansion of the progenitor pools actually occurred in these mice. The IUE-mediated GFP labeling of RGCs lining the apical surface in the E14.5 *Rapgef2*-cKO embryos largely confirmed the above observation; at E16.5 both Pax6^+^/GFP^+^ cells and Tbr2^+^/GFP^+^ cells almost completely disappeared from the VZ, and were mainly located in the IZ and CP (Fig. 6*B*). Preservation of progenitor properties in the ectopically located Pax6^+^ or Tbr2^+^ cells was further supported by fate analysis of BrdU-incorporated cells, which showed that these cells could differentiate into neurons constituting the neocortex and ECM in postnatal *Rapgef2*-cKO mice (Fig. 8). These results were consistent with the substantial increase in the total numbers of late-born neurons in *Rapgef2*-cKO mice, and in both early-born and late-born neurons in *Rapgef2/6*-dKO mice at P90.

As for the mechanism for the aberrant progenitor localization, we found that the apical surfaces lining the ventricle exhibited irregular morphology and that a dense array of the AJs on the apical surfaces became almost nonexistent in the *Rapgef2*-cKO cortices at E15.5 and the *Rapgef2/6*-dKO cortices at E13.5 and E15.5 (Figs. 3*B*, 4). The IUE-mediated GFP labeling of RGCs in the E14.5 *Rapgef2*-cKO embryos largely confirmed the observations made with the E15.5 *Rapgef2*-cKO cortices (Fig. 2); at E16.5, the apical surface exhibited very irregular cell arrangements, and not only the apical fiber end feet and their AJs, but also the apical fibers themselves of Pax6^+^/GFP^+^ cells became almost nonexistent (Fig. 6*D*). Thus, the extents of the structural disturbance of the apical surface structure apparently correlated with those of the aberrant localization of neural progenitors. The difference in the severity of the various phenotypes between *Rapgef2*-cKO and *Rapgef2/6*-dKO mice seemed to be accounted for by the temporal difference in the expression of Rapgef2 and Rapgef6; both Rapgef2 and Rapgef6 at E13.5, but Rapgef2 only at E15.5, were expressed and concentrated on the apical surface (Fig. 3). Thus, Rapgef6 was capable of compensating for the loss of Rapgef2 function at E13.5, but not at 15.5, which could account for the differential involvements of early-born and late-born neurons in the ECM formation between *Rapgef2*-cKO and *Rapgef2/6*-dKO mice (Fig. 1).

Analysis of the cell-autonomous effect of *Rapgef2* deletion through IUE-mediated cotransduction of Cre recombinase and EGFP into the E14.5 brains revealed that at E16.5 the proportion of Pax6^+^/GFP^+^ cells showed a marked decrease in the VZ, which was accompanied by an increase in the proportion of GFP^+^ cells, though it was totally negative for Pax6 or Tbr2, located in the IZ, suggesting earlier detachment of RGCs from the apical surface (Fig. 6*B*,*C*). Moreover, the numbers of GFP^+^ apical fibers and their end feet on the apical surface were substantially reduced, and the apical surface AJs were compromised at E16.5, which was similar to the observation with the *Rapgef2*-cKO cortices (Fig. 6*D*). Intriguingly, these aberrant phenotypes were effectively suppressed by the coexpression of the constitutively active Rap1 mutant Rap1^G12V^ (Fig. 6*E–G*). These results indicated that Rapgef2 possesses a cell-autonomous function, governed by its Rap1-GEF activity, in maintaining the apical surface AJ structures and preventing earlier detachment of RGCs from the apical surface. However, a non-cell-autonomous effect of the *Rapgef2* knockout seems to be responsible for the preservation of the progenitor properties in RGCs and IPCs detached from the apical surface, as observed in the *Rapgef2*-cKO cortices. Recent studies showed that loss of the attachment of RGCs to the apical surface, such as that induced by shRNA-mediated knockdown of N-cadherin, led to reduction of β-catenin signaling, premature neuronal differentiation, cell cycle exit and increased migration toward the CP, suggesting that earlier detachment of RGCs from the apical surface might lead to premature neuronal differentiation (Zhang et al., 2010, 2013). Presently, we have no idea of accounting for the preservation of the neural progenitor properties in RGCs and IPCs of *Rapgef2*-cKO embryos, which are detached from the apical surface and ectopically located. In this sense, it may be noteworthy to point out that the phenotype of ectopic localization of RGCs while retaining the progenitor properties was also observed in mice with knockout of the AJ proteins such as N-cadherin, E-cadherin, and afadin (Machon et al., 2003; Lien et al., 2006; Kadowaki et al., 2007; Gil-Sanz et al., 2014; Yamamoto et al., 2015).

We also observed a defect in the multipolar–bipolar transition of neurons in *Rapgef2*-cKO embryos by IUE-mediated GFP labeling of RGCs at E14.5, confirming the previous report (Ye et al., 2014). Almost all of the GFP^+^ cells, which were also Cux1^+^, failed to reach layers II–IV and were located in the ECM of the *Rapgef2*-cKO cortex at P7, and about half of them assumed multipolar morphology (Fig. 7). The ectopic accumulation of neurons observed at E15.5 was likely to be a predecessor of the ECM formed in the adult *Rapgef2*-cKO and *Rapgef2*/*6*-dKO cortices. However, analysis of the cell-autonomous effect of *Rapgef2* deletion through IUE-mediated cotransduction of Cre recombinase and EGFP into the E14.5 brains revealed that almost all of the Cux1^+^/GFP^+^ neurons successfully reached layers II–IV at P7. The Cux1^+^/GFP^+^ neurons exhibited multipolar morphology in a high proportion and a significant delay in migration at E18.5, indicating the existence of a multipolar–bipolar transition defect. These results suggested that the loss of the Rapgef2 function caused transient defects in the migration and multipolar–bipolar transition of neurons in a cell-autonomous manner. On the other hand, further contribution of a non-cell-autonomous effect, presumably implemented by Rapgef2-deficient surroundings of the migrating neurons, was needed to yield the corresponding defects in the *Rapgef2*-cKO cortices, which lasted to adulthood and led to the ECM formation. In this line, the disorganization of the RG fiber system observed in the *Rapgef2*-cKO cortices may play a key role by compromising neuronal migration dependent on its guidance. Indeed, a certain correlation was observed between the extent of RG fiber disorganization and the migration defect of Ctip2^+^ neurons (Fig. 5). However, we presently do not know how neurons produced from the ectopically located neural progenitors migrate and form the apparently normal neocortex and the ECM, as suggested by findings of the BrdU labeling experiment (Fig. 8).

During the preparation of this article, analysis of brain malformations in mice lacking both Rap1a and Rap1b in their cerebral cortices was reported (Shah et al., 2016). The cerebral cortices of these mice exhibited severe developmental defects similar to those observed in *Rapgef2*-cKO and *Rapgef2/6*-dKO mice in our study, which were characterized by ectopic localization of neural progenitors, disruption of the AJs in the VZ, and impairment of the multipolar–bipolar transition. These results demonstrate the crucial and predominant role of Rapgef2 and Rapgef6 in the formation of the apical surface AJ structure, which is essential for the proper localization of neural progenitors, and the multipolar–bipolar transition of neurons via mediating Rap1 activation downstream of presently unknown extracellular stimuli. It may be noteworthy that rare inherited copy number variations of the *RAPGEF6* gene, involving microdeletions of its exons 2-11, were reported to exhibit strong genetic association with schizophrenia (Xu et al., 2008). A recent study (Levy et al., 2015) showed that *Rapgef6* knock-out mice exhibited impaired function of the amygdala and hippocampus, brain regions that are implicated in schizophrenia pathophysiology. Moreover, a chromosomal region containing the *RAPGEF2* gene was identified as a rare inherited copy number variant with a linkage to schizophrenia (Xu et al., 2009). Further studies on Rapgef2 and Rapgef6 will help in advancing our understanding of not only the fundamental mechanism for the cerebral corticogenesis but also the etiology of various CNS diseases.

## Synthesis

The decision was a result of the Reviewing Editor Orly Reiner and the peer reviewers coming together and discussing their recommendations until a consensus was reached. A fact-based synthesis statement explaining their decision and outlining what is needed to prepare a revision is listed below. The following reviewers agreed to reveal their identity: NONE.

## References

[B1] Bilasy SE, Satoh T, Ueda S, Wei P, Kanemura H, Aiba A, Terashima T, Kataoka T (2009) Dorsal telencephalon-specific RA-GEF-1 knockout mice develop heterotopic cortical mass and commissural fiber defect. Eur J Neurosci 29:1994–2008. 10.1111/j.1460-9568.2009.06754.x [TQ1] 19453629

[B2] Bilasy SE, Satoh T, Terashima T, Kataoka T (2011) RA-GEF-1 (Rapgef2) is essential for proper development of the midline commissures. Neurosci Res 71:200–209. 10.1016/j.neures.2011.08.004 21864586

[B3] Boettner B, Van Aelst L (2009) Control of cell adhesion dynamics by Rap1 signaling. Curr Opin Cell Biol 21:684–693. 10.1016/j.ceb.2009.06.004 19615876 PMC2841981

[B4] Chen Y, Wang PY, Ghosh A (2005) Regulation of cortical dendrite development by Rap1 signaling. Mol Cell Neurosci 28:215–228 10.1016/j.mcn.2004.08.012 15691704

[B5] Defelipe J (2011) The evolution of the brain, the human nature of cortical circuits, and intellectual creativity. Front Neuroanat 5:29 10.3389/fnana.2011.00029 21647212 PMC3098448

[B6] de Rooij J, Boenink NM, van Triest M, Cool RH, Wittinghofer A, Bos JL (1999) PDZ-GEF1, a guanine nucleotide exchange factor specific for Rap1 and Rap2. J Biol Chem 274:38125–38130. 10608883 10.1074/jbc.274.53.38125

[B7] Englund C, Fink A, Lau C, Pham D, Daza RA, Bulfone A, Kowalczyk T, Hevner RF (2005) Pax6, Tbr2, and Tbr1 are expressed sequentially by radial glia, intermediate progenitor cells, and postmitotic neurons in developing neocortex. J Neurosci 25:247–251. 10.1523/JNEUROSCI.2899-2804.2005 15634788 PMC6725189

[B8] Franco SJ, Martinez-Garay I, Gil-Sanz C, Harkins-Perry SR, Müller U (2011) Reelin regulates cadherin function via Dab1/Rap1 to control neuronal migration and lamination in the neocortex. Neuron 69:482–497. 10.1016/j.neuron.2011.01.003 21315259 PMC3056352

[B9] Gao X, Satoh T, Liao Y, Song C, Hu CD, Kariya Ki K, Kataoka T (2001) Identification and characterization of RA-GEF-2, a Rap guanine nucleotide exchange factor that serves as a downstream target of M-Ras. J Biol Chem 276:42219–42225. 10.1074/jbc.M105760200 [Mismatch] 11524421

[B10] Gil-Sanz C, Landeira B, Ramos C, Costa MR, Müller U (2014) Proliferative defects and formation of a double cortex in mice lacking Mltt4 and Cdh2 in the dorsal telencephalon. J Neurosci 34:10475–10487. 10.1523/JNEUROSCI.1793-1714.2014 25100583 PMC4200106

[B11] Gloerich M, Bos JL (2011) Regulating Rap small G-proteins in time and space. Trends Cell Biol 21:615–623. 10.1016/j.tcb.2011.07.001 21820312

[B12] Götz M, Huttner WB (2005) The cell biology of neurogenesis. Nat Rev Mol Cell Biol 6:777–788. 10.1038/nrm1739 16314867

[B13] Greig LC, Woodworth MB, Galazo MJ, Padmanabhan H, Macklis JD (2013) Molecular logic of neocortical projection neuron specification, development and diversity. Nat Rev Neurosci 14:755–769. 10.1038/nrn3586 24105342 PMC3876965

[B14] Hoerder-Suabedissen A, Molnár Z (2015) Development, evolution and pathology of neocortical subplate neurons. Nat Rev Neurosci 16:133–146. 10.1038/nrn3915 25697157

[B15] Junghans D, Hack I, Frotscher M, Taylor V, Kemler R (2005) Beta-catenin-mediated cell-adhesion is vital for embryonic forebrain development. Dev Dyn 233:528–539. 10.1002/dvdy.20365 15844200

[B16] Jossin Y, Cooper JA (2011) Reelin, Rap1 and N-cadherin orient the migration of multipolar neurons in the developing neocortex. Nat Neurosci 14:697–703. 10.1038/nn.2816 21516100 PMC3102785

[B17] Kadowaki M, Nakamura S, Machon O, Krauss S, Radice GL, Takeichi M (2007) N-cadherin mediates cortical organization in the mouse brain. Dev Biol 304:22–33. 10.1016/j.ydbio.2006.12.014 17222817

[B18] Kawauchi T, Chihama K, Nishimura YV, Nabeshima Y, Hoshino M (2005) MAP1B phosphorylation is differentially regulated by Cdk5/p35, Cdk5/p25, and JNK. Biochem Biophys Res Commun 331:50–55. 10.1016/j.bbrc.2005.03.132 15845356

[B19] Levy RJ, Kvajo M, Li Y, Tsvetkov E, Dong W, Yoshikawa Y, Kataoka T, Bolshakov VY, Karayiorgou M, Gogos JA (2015) Deletion of Rapgef6, a candidate schizophrenia susceptibility gene, disrupts amygdala function in mice. Transl Psychiatry 5:e577 10.1038/tp.2015.75 26057047 PMC4490285

[B20] Liao Y, Kariya K, Hu CD, Shibatohge M, Goshima M, Okada T, Watari Y, Gao X, Jin TG, Yamawaki-Kataoka Y, Kataoka T (1999) RA-GEF, a novel Rap1A guanine nucleotide exchange factor containing a Ras/Rap1A-associating domain, is conserved between nematode and humans. J Biol Chem 274:37815–37820. 10608844 10.1074/jbc.274.53.37815

[B21] Liao Y, Satoh T, Gao X, Jin TG, Hu CD, Kataoka T (2001) RA-GEF-1, a guanine nucleotide exchange factor for Rap1, is activated by translocation induced by association with Rap1·GTP and enhances Rap1-dependent B-Raf activation. J Biol Chem 276:28478–28483. 10.1074/jbc.M101737200 11359771

[B22] Lien WH, Klezovitch O, Fernandez TE, Delrow J, Vasioukhin V (2006) alphaE-catenin controls cerebral cortical size by regulating the hedgehog signaling pathway. Science 311:1609–1612. 10.1126/science.1121449 16543460 PMC2556178

[B23] Machon O, van den Bout CJ, Backman M, Kemler R, Krauss S (2003) Role of beta-catenin in the developing cortical and hippocampal neuroepithelium. Neuroscience 122:129–143. 14596855 10.1016/s0306-4522(03)00519-0

[B24] Nagano T, Edamatsu H, Kobayashi K, Takenaka N, Yamamoto M, Sasaki N, Nishimura Y, Kataoka T (2014) Phospholipase Cε, an effector of Ras and Rap small GTPases, is required for airway inflammatory response in a mouse model of bronchial asthma. PLoS One 9:e108373 10.1371/journal.pone.0108373 25269075 PMC4182471

[B25] Nishimura YV, Shikanai M, Hoshino M, Ohshima T, Nabeshima Y, Mizutani K, Nagata K, Nakajima K, Kawauchi T (2014) Cdk5 and its substrates, Dcx and p27kip1, regulate cytoplasmic dilation formation and nuclear elongation in migrating neurons. Development 141:3540–3550. 10.1242/dev.111294 25183872

[B26] Saito T, Nakatsuji N (2001) Efficient gene transfer into the embryonic mouse brain using in vivo electroporation. Dev Biol 240:237–246. 10.1006/dbio.2001.0439 11784059

[B27] Schmid MT, Weinandy F, Wilsch-Bräuninger M, Huttner WB, Cappello S, Götz M (2014) The role of α-E-catenin in cerebral cortex development: radial glia specific effect on neuronal migration. Front Cell Neurosci 8:215 10.3389/fncel.2014.00215 25147501 PMC4124588

[B28] Sekine K, Kawauchi T, Kubo K, Honda T, Herz J, Hattori M, Kinashi T, Nakajima K (2012) Reelin controls neuronal positioning by promoting cell-matrix adhesion via inside-out activation of integrin α5β1. Neuron 76:353–369. 10.1016/j.neuron.2012.07.020 23083738 PMC3479437

[B29] Shah B, Lutter D, Tsytsyura Y, Glyvuk N, Sakakibara A, Klingauf J, Püschel AW (2016) Rap1 GTPases are master regulators of neural cell polarity in the developing neocortex. Cereb Cortex. Advance online publication. Retrieved June 12, 2016. doi:10.1093/cercor/bhv341. 10.1093/cercor/bhv341 26733533

[B30] Shitamukai A, Konno D, Matsuzaki F (2011) Oblique radial glial divisions in the developing mouse neocortex induce self-renewing progenitors outside the germinal zone that resemble primate outer subventricular zone progenitors. J Neurosci 31:3683–3695. 10.1523/JNEUROSCI.4773-4710.2011 21389223 PMC6622781

[B31] Srivastava DP, Jones KA, Woolfrey KM, Burgdorf J, Russell TA, Kalmbach A, Lee H, Yang C, Bradberry MM, Wokosin D, Moskal JR, Casanova MF, Waters J, Penzes P (2012) Social, communication, and cortical structural impairments in Epac2-deficient mice. J Neurosci 32:11864–11878. 10.1523/JNEUROSCI.1349-1312.2012 22915127 PMC3520089

[B32] Tsumura H, Yoshida T, Saito H, Imanaka-Yoshida K, Suzuki N (2006) Cooperation of oncogenic K-ras and p53 deficiency in pleomorphic rhabdomyosarcoma development in adult mice. Oncogene 25:7673–7679. 10.1038/sj.onc.1209749 16785989

[B33] Voss AK, Britto JM, Dixon MP, Sheikh BN, Collin C, Tan SS, Thomas T (2008) C3G regulates cortical neuron migration, preplate splitting and radial glial cell attachment. Development 135:2139–2149. 10.1242/dev.016725 18506028

[B34] Wei P, Satoh T, Edamatsu H, Aiba A, Setsu T, Terashima T, Kitazawa S, Nakao K, Yoshikawa Y, Tamada M, Kataoka T (2007) Defective vascular morphogenesis and mid-gestation embryonic death in mice lacking RA-GEF-1. Biochem Biophys Res Commun 363:106–112. 10.1016/j.bbrc.2007.08.149 17826737

[B35] Xie Z, Huganir RL, Penzes P (2005) Activity-dependent dendritic spine structural plasticity is regulated by small GTPase Rap1 and its target AF-6. Neuron 48:605–618. 10.1016/j.neuron.2005.09.027 16301177

[B36] Xu B, Roos JL, Levy S, van Rensburg EJ, Gogos JA, Karayiorgou M (2008) Strong association of de novo copy number mutations with sporadic schizophrenia. Nat Genet 40:880–885. 10.1038/ng.162 18511947

[B37] Xu B, Woodroffe A, Rodriguez-Murillo L, Roos JL, van Rensburg EJ, Abecasis GR, Gogos JA, Karayiorgou M (2009) Elucidating the genetic architecture of familial schizophrenia using rare copy number variant and linkage scans. Proc Natl Acad Sci U S A 106:16746–16751. 10.1073/pnas.0908584106 19805367 PMC2757863

[B38] Yamamoto H, Mandai K, Konno D, Maruo T, Matsuzaki F, Takai Y (2015) Impairment of radial glial scaffold-dependent neuronal migration and formation of double cortex by genetic ablation of afadin. Brain Res 1620:139–152. S0006-8993(15)00397-3972. 25988834 10.1016/j.brainres.2015.05.012

[B39] Ye T, Ip JP, Fu AK, Ip NY (2014) Cdk5-mediated phosphorylation of RapGEF2 controls neuronal migration in the developing cerebral cortex. Nat Commun 5:4826 10.1038/ncomms5826 25189171 PMC4164783

[B40] Yoshikawa Y, Satoh T, Tamura T, Wei P, Bilasy SE, Edamatsu H, Aiba A, Kataoka T (2007) The M-Ras-RA-GEF-2-Rap1 pathway mediates tumor necrosis factor-alpha dependent regulation of integrin activation in splenocytes. Mol Biol Cell 18:2949–2959. 10.1091/mbc.E07-03-0250 17538012 PMC1949361

[B41] Zhang J, Woodhead GJ, Swaminathan SK, Noles SR, McQuinn ER, Pisarek AJ, Stocker AM, Mutch CA, Funatsu N, Chenn A (2010) Cortical neural precursors inhibit their own differentiation via N-cadherin maintenance of beta-catenin signaling. Dev Cell 18:472–479. 10.1016/j.devcel.2009.12.025 20230753 PMC2865854

[B42] Zhang J, Shemezis JR, McQuinn ER, Wang J, Sverdlov M, Chenn A (2013) AKT activation by N-cadherin regulates beta-catenin signaling and neuronal differentiation during cortical development. Neural Dev 8:7 10.1186/1749-8104-8-7 23618343 PMC3658902

